# Liver changes in severe *Plasmodium falciparum* malaria: histopathology, apoptosis and nuclear factor kappa B expression

**DOI:** 10.1186/1475-2875-13-106

**Published:** 2014-03-17

**Authors:** Parnpen Viriyavejakul, Vasant Khachonsaksumet, Chuchard Punsawad

**Affiliations:** 1 Department of Tropical Pathology, Faculty of Tropical Medicine, Mahidol University, 420/6 Rajvithi Road, Bangkok 10400, Thailand; 2 Center for Emerging and Neglected Infectious Diseases, Mahidol University, Salaya, Nakhon Pathom 73170, Thailand; 3 School of Medicine, Walailak University, 222, Thasala District, Nakhon Si Thammarat 80161, Thailand

**Keywords:** Malaria, *Plasmodium falciparum*, Liver, Kupffer cells, Bilirubin, Nuclear factor kappa B, NF-κB p65, Cleaved caspase-3, Apoptosis, Immunohistochemistry

## Abstract

**Background:**

Liver involvement in severe *Plasmodium falciparum* infection is commonly a significant cause of morbidity and mortality among humans. The clinical presentation of jaundice often reflects a certain degree of liver damage. This study investigated the liver pathology of severe *P. falciparum* malaria as well as the regulation and occurrence of apoptosis in cellular components of formalin-fixed, paraffin-embedded liver tissues.

**Methods:**

The liver tissues used in the study came from patients who died from *P. falciparum* malaria with hyperbilirubinaemia (total bilirubin (TB) ≥ 51.3 μmol/L or 3 mg/dl) (12 cases), *P. falciparum* malaria without hyperbilirubinaemia (TB < 51.3 μmol/L) (10 cases); and patients who died due to accidents, whose liver histology was normal (the control group) (10 cases). The histopathology of the liver tissue was studied by routine histology method. Caspase-3 and nuclear factor kappa B (NF-κB) p65 expressions were determined using immunohistochemistry.

**Results:**

The severity of liver histopathology, occurrence of apoptosis and NF-κB p65 activation in *P. falciparum* malaria were associated with higher TB level. Significant correlations were found between NF-κB p65 expression and apoptosis in Kupffer cells and lymphocytes in the portal tracts.

**Conclusions:**

Hyperplastic Kupffer cells and portal tract inflammation are two main features found in the liver tissues of severe *P. falciparum* malaria cases. In addition, NF-κB is associated with Kupffer cells and lymphocyte apoptosis in severe *P. falciparum* malaria.

## Background

*Plasmodium falciparum* malaria is a life-threatening infectious disease that remains a major global health problem. The severe manifestations often present clinically as cerebral malaria, pulmonary oedema, acute kidney injury, hypoglycaemia, lactic acidosis, anaemia and liver involvement [1]. *Plasmodium falciparum* malaria causes clinical jaundice in 2.5-5.3% of cases in endemic areas [2,3]. The liver is an important organ involved during the hepatic stage of the malaria parasite’s life cycle, where malaria sporozoites develop into merozoites. The merozoites are then released into the circulation and enter the erythrocytic stage. In the erythrocytic stage, parasitized red blood cells (PRBCs) become sequestered in small blood vessels. The degraded haemozoin pigment is then engulfed by local tissue macrophages, such as Kupffer cells and alveolar macrophages. Common histopathological findings of the liver in *P. falciparum* malaria include reactive Kupffer cells, retention of haemozoin pigment and minimal PRBC sequestration [4,5]. An ultrastructural study reported an association between high PRBC load in the livers of malaria patients with jaundice, hepatomegaly and liver enzyme elevation [6].

Apoptotic changes occur in a variety of cellular systems and involve both physiologic and pathologic changes. While apoptotic change in the liver have not been documented in human malaria, changes have been reported in animal models during the erythrocytic stage in hepatocytes [7,8] and during the hepatic stage in Kupffer cells [9]. This process of programmed cell death can be mediated by various stimuli, including hormones, cytokines, growth factors, bacterial or viral infections and the immune response [10]. Cell apoptosis is regulated via two major pathways: the intrinsic or mitochondrial pathway and the extrinsic or death-receptor pathway. Initiator caspases, such as caspase-8 or -9, play a regulatory role by activating downstream effector caspases, such as caspase-3, -6, or -7 [11]. NF-κB has been shown to regulate the apoptotic program in various cell types, either as an up-regulating response or as an apoptosis blocker [12]. Evidence of NF-κB regulating apoptosis was found in the brain endothelial cells and intravascular lymphocytes in cerebral malaria [13]. However, no linkage between NF-κB and apoptosis has been reported in the livers of *P. falciparum* malaria patients. This study evaluated the liver pathology of severe *P. falciparum* malaria in association with total bilirubin (TB) level. The occurrence of apoptosis and its relation to a signaling molecule (NF-κB) in liver tissues was investigated.

## Methods

### Liver tissue specimens from malaria patients and controls

Specimens were classified into two groups according to the level of total bilirubin (TB): TB ≥ 51.3 μmol/L (3 mg/dl) (12 cases) and TB < 51.3 μmol/L (10 cases), based on laboratory data. Liver specimens with normal histology obtained from fatal accident cases (10 cases), served as the control group. The specimens were obtained from autopsied cases at the Department of Tropical Pathology, Faculty of Tropical Medicine, Mahidol University, Bangkok, Thailand. Control cases came from the same region. Patients with hepatitis B co-infection, and patients with glucose-6-phosphate dehydrogenase (G6PD) deficiency, were excluded from the study. The use of left-over liver specimens and the study protocol were reviewed and approved by the Ethics Committee of the Faculty of Tropical Medicine, Mahidol University (MUTM 2011-025-01 and MUTM 2011-025-02).

### Liver tissue preparation

Left-over liver tissues in paraffin-embedded blocks were re-embedded with new paraffin medium using standard histological techniques. Liver tissues were sectioned at 4 μm thickness and placed onto glass slides for histopathological examination and onto positively charged slides for immunohistochemistry study against cleaved caspase-3 and NF-κB p65.

### Histology of liver in severe *P. falciparum* malaria

Overall liver histopathology was classified based on the severity of six histological criteria, namely fatty change, hyperplastic Kupffer cells, portal tract inflammation, bile duct proliferation, sinusoidal congestion and haemozoin deposition. Quantification was performed under high (400x) magnification. The severity level of each histopathological change was graded on a scale from 0 to 3, according to semi-quantitative assessment (Table 1). The highest possible total score was 18 (6 histological criteria x 3 as highest scale). Score 0 meant no histopathological change and score 18 referred to most severe histopathological change. Microscopic evaluation was done by two observers (PV and CP). A third person was asked to score the histopathological changes if more than 2 grading variations were observed.

**Table 1:** Histopathological changes and grading schemes used to evaluate liver tissues

**Histopathologic changes**	**Histopathologic grading**			
	**0**	**1**	**2**	**3**
Fatty change	No fatty change	< 10%	10-50%	> 50%
Kupffer cells/HPF	< 20/HPF	20-35/HPF	36-50/HPF	> 50/HPF
Portal tract inflammation	< 5% of portal tract area	5-15% of portal tract area	16-30% of portal tract area	> 30% of portal tract area
Bile duct proliferation	No proliferation	Mild proliferation	Moderate proliferation	Severe proliferation
Sinusoid congestion	No congestion	Mild congestion	Moderate congestion	Severe congestion
Haemozoin deposition	No deposition	Mild deposition	Moderate deposition	Severe deposition

HPF = high power field (magnification 400x).

### Immunohistochemical staining of cleaved caspase-3 and NF-κB p65

The 4 μm paraffin sections were deparaffinized and rehydrated by sequential immersion in a graded series of alcohol, then transferred into water for 5 mins. To inhibit endogenous peroxidase activity, the sections were incubated with 3% H_2_O_2_ for 5 mins. The sections were then heated in a microwave oven (in 0.1 M sodium citrate buffer, pH 6.0 for cleaved caspase-3 and 0.1 M Tris–HCl buffer, pH 9.0 for NF-κB p65) for 20 mins for epitope retrieval. After washing with phosphate buffered saline (PBS), pH 7.4, sections were incubated for 30 mins with normal serum as blocking solution to reduce the non-specific background, then cooled to room temperature while still immersed in buffer. The following protocol was realized using avidin-biotin alkaline phosphatase complex (VECTASTAIN® ABC-AP kit (Rabbit IgG) # AK-5001) for cleaved caspase 3 and avidin-biotin peroxidase complex (VECTASTAIN® ABC kit (Mouse IgG) # PK-4002) for NF-κB p65 (Vector Laboratories, Inc., USA) according to the manufacturer’s protocol. The sections were incubated with primary antibody; rabbit polyclonal anti-cleaved caspase-3 (Asp175) antibody (1:200 dilution) (Cell Signaling Technology, USA) and mouse monoclonal anti-NF-κB p65 (1:50 dilution) (Santa Cruz Biotechnology Inc., Santa Cruz, CA) and incubated overnight at 4˚C in a humidity chamber. The following day, sections were washed three times with PBS, and incubated with anti-mouse/rabbit biotinylated secondary antibody (Vector Laboratories, Inc., USA) for 30 mins at room temperature and reacted with avidin-biotin complex (ABC) conjugated with alkaline phosphatase (AP)/horseradish peroxidase (HRP) (Vector Laboratories, Inc., USA) for 30 mins. After washing, enzyme activity was visualized by Vector® Red substrate kit (Vector Laboratories, Inc., USA) for cleaved caspase-3, resulting in the formation of a red colour at the antigen sites and by 3,3′- diaminobenzidine (DAB) (Vector Laboratories, Inc., USA) for NF-κB p65, presenting as a brown colour. Subsequently, sections were counterstained with haematoxylin for 1 min, and mounted with a coverslip.

### Cleaved caspase-3 and NF-κB p65 analysis

The presence of immunopositive cells for cleaved caspase-3 and NF-κB p65 was recorded as percentages. In addition, immunostaining intensity was scored from 0–3 (0-negative staining, 1- mild, 2- moderate, and 3- strong immunostaining). Total score was calculated by multiplying percentage immunopositive cells and intensity, a method used by Punsawad *et al*. 2013 [13].

### Measurement of Kupffer cell length

Ten representative views of H&E-stained liver sections were randomly photographed at 400x magnification using an Olympus Bx41 light microscope (Olympus, Tokyo, Japan) connected to an Olympus DP20 digital camera (Olympus, Tokyo, Japan). Kupffer cell length was measured with the UTHSCSA Image Tool program (developed at the University of Texas Health Science Center at San Antonio, TX; freely available from the Internet).

### Statistical analysis

Data were expressed as mean ± standard error of the mean (SEM). The normality of distribution was determined by the Kolmogorov-Smirnov test. Differences between groups were analyzed by Mann Whitney *U-*test. In addition, the correlations of each variable within groups and pertinent clinical data were calculated using Spearman’s rank correlation (*r*_*s*_). Statistical analysis was performed using SPSS version 17.0 software (SPSS, IL, USA). A *p* value < 0.05 was considered significantly different.

## Results

### Malaria patients

Twenty-two liver specimens were collected from *P. falciparum* malaria cases, consisting of 12 cases with hyperbilirubinaemia (total bilirubin (TB) ≥ 51.3 μmol/L or 3 mg/dl) and 10 cases without hyperbilirubinaemia (TB < 51.3 μmol/L). Ten cases with normal liver histopathology served as controls. Table 2 summarizes the mean age, sex, days of fever pre-admission, parasitaemia and important liver function tests of the malaria patients. Significant differences between two groups were noted in the levels of albumin, aspartate aminotransferase (AST), alanine aminotransferase (ALT), alkaline phosphatase, total and direct bilirubin (*p* value < 0.05). Common associated complications were cerebral malaria (70.00% for non-hyperbilirubinaemia and 33.33% for hyperbilirubinaemia groups) and acute kidney injury (33.33% for hyperbilirubinaemia group).

**Table 2:** **Clinical and laboratory parameters of *P. falciparum *** **malaria patients**

	**Non-hyperbilirubinaemia (TB < 51.3 μmol/L) (n = 10)**	**Hyperbilirubinaemia (TB ≥ 51.3 μmol/L) (n = 12)**
Age (years) (*p* = 0.974)	25.8 ± 3.95	26.17 ± 4.28
Sex (M:F)	6:4	10:2
Days of fever (*p* = 0.095)	4.1 ± 0.82	5.83 ± 0.77
Parasitaemia (/μl) (*p* = 0.619)	303,186.67 ± 151,070.30	391,501.40 ± 183,362.30
Albumin (g/L) (*p* = 0.006)	33.2 ± 0.17	25.3 ± 0.13
AST (U/L) (*p* = 0.006)	72.00 ± 21.41	266.33 ± 64.93
ALT (U/L) (*p* = 0.049)	49.17 ± 8.23	126.79 ± 29.22
Alkaline phosphatase (U/L) (*p* = 0.049)	8.42 ± 2.62	20.07 ± 4.33
Total bilirubin (μmol/L) (*p* = 0.001)	30.61 ± 0.31	441.60 ± 5.39
Direct bilirubin (μmol/L) (*p* = 0.001)	8.55 ± 0.15	217.17 ± 2.73

### Histopathology of liver in severe *P. falciparum* malaria

**Figure 1:**
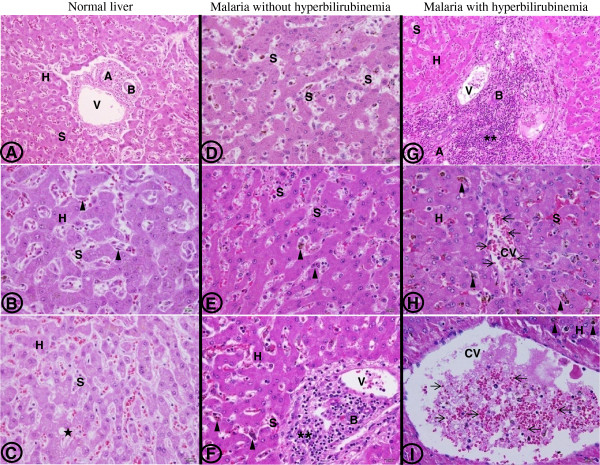
**Histopathological changes of liver tissue from normal controls (A-C), from severe *P. falciparum *malaria patients without hyperbilirubinaemia (D-F) and from severe *P. falciparum *malaria patients with hyperbilirubinaemia (G-I).** Images A and G are shown at 200x magnification; bars are 50 μm. Other images are at 400x magnification; bars are 20 μm. H– hepatocytes, A- hepatic artery, V- hepatic vein, B- bile duct, S- sinusoidal area, CV- central vein, arrowheads- Kupffer cells, star- fatty change, asterisks- inflammatory cells, arrows- PRBCs.

Figure 1 displays histopathological changes of the liver in severe *P. falciparum* malaria. Morphologically, hepatocytes and endothelial cells (ECs) in the liver were generally unaffected in severe *P. falciparum* malaria. Fatty change and bile duct proliferation were not features of *P. falciparum* infection. Hyperplastic Kupffer cells, portal tract inflammation, sinusoidal congestion and haemozoin pigment deposition were important pathological hallmarks related to higher TB levels (Table 3). The total histopathological grading score showed the overall changes and was found to be highest among the group of malaria patients with hyperbilirubinaemia (12/18). Figure 1 (A-C) illustrates a normal portal tract, consisting of hepatic vein, hepatic artery, and bile duct, with surrounding hepatocytes. Very few inflammatory cells are noted within the portal tract. Minimal fatty change is sometimes observed (Figure 1C). Liver tissues from malaria patient without hyperbilirubinaemia show enlarged sinusoidal area, haemozoin pigment within the hyperplastic Kupffer cells, and inflammatory cells within the portal tract (Figure 1, D-F). In hyperbilirubinaemia group (Figure 1, G-I), liver tissues show dense inflammatory cells infiltration in the portal tract. At higher magnification, hyperplastic Kupffer cells are visible and contain haemozoin pigment. Sinusoidal areas are often congested. Central vein contains numerous PRBCs.

**Table 3:** **Histopathologic grading of liver tissues in *P. falciparum *** **malaria**

**Histopathologic changes**	**Histopathologic grading**		
	**Normal liver**	**Non-hyperbilirubinaemia (TB < 51.3 μmol/L)**	**Hyperbilirubinaemia (TB ≥ 51.3 μmol/L)**
Fatty change	0	0	0
Kupffer cells hyperplasia	0	1	3
Portal tract inflammation	1	2	3
Bile duct proliferation	0	0	0
Sinusoid congestion	0	2	3
Haemozoin deposition	0	3	3
Total histological score	1/18	8/18	12/18

**Figure 2:**
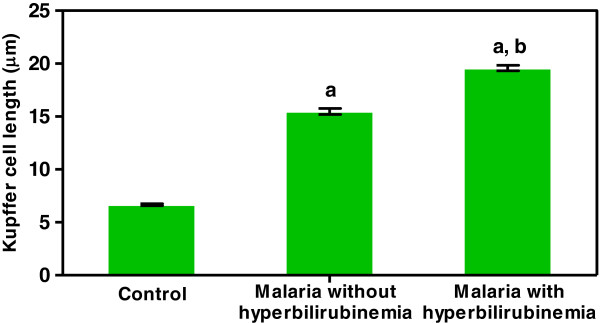
**Length of Kupffer cells as measured and analyzed by Image Tool software.** Significant differences in Kupffer cell length were observed between the severe malaria groups (with and without hyperbilirubinaemia) and the normal controls (a) and between the hyperbilirubinaemia and non-hyperbilirubinaemia groups (b) (*p* < 0.05). Data are presented as a mean ± SEM.

Fatty change, hyperplastic Kupffer cells and portal tract inflammation were quantified in each group (Table 4). There was no significant difference between fatty changes in the livers of the *P. falciparum* malaria groups and the normal controls (all *p* > 0.05). However, hyperplastic Kupffer cells and portal tract inflammation were significantly higher in the malaria groups compared with the normal controls and highest in the livers of the *P. falciparum* patients with hyperbilirubinaemia. The number of hyperplastic Kupffer cells were increased in the malaria group with hyperbilirubinaemia (52.21 ± 2.32/high power field (HPF)), compared with the normal liver group (9.65 ± 0.67/HPF) (*p* < 0.001) and the malaria group without hyperbilirubinaemia (32.05 ± 3.34/HPF) (*p* < 0.001). Generally, Kupffer cell contains packed haemozoin pigment within the cytoplasm. Positive correlations were evident between TB and the number of Kupffer cells (*r*_*s*_ = 0.551, *p* = 0.018) and between TB and % lymphocytes in the portal tract (*r*_*s*_ = 0.743. *p* = 0.020). In terms of Kupffer-cell length (Figure 2), the size of Kupffer cells as measured and analysed by Image Tool software showed significant numbers of reactive Kupffer cells in the malaria group with hyperbilirubinaemia (more than three times), compared with the normal liver group (*p* < 0.001) and the non-hyperbilirubinaemia group (*p* < 0.001).

**Table 4:** **Quantification of fatty changes, hyperplastic Kupffer cells and portal inflammation in the livers of *P. falciparum *** **malaria patients compared with the control group**

**Histopathological changes**	**Normal liver (n = 10)**	**Non-hyperbilirubinaemia (TB < 51.3 μmol/L) (n = 10)**	**Hyperbilirubinaemia (TB ≥ 51.3 μmol/L) (n = 12)**
Fatty changes (%/HPF)	0.59 ± 0.25	1.71 ± 0.46	1.97 ± 0.67
Hyperplastic Kupffer cells (count/HPF)	9.65 ± 0.67	32.05 ± 3.34^a^	52.21 ± 2.32^a,b^
Portal tract inflammation (%)	5.49 ± 1.20	17.1 ± 1.62^a^	32.79 ± 2.48^a,b^

^a^ Significant difference compared with normal controls, ^b^ Significant difference between malaria groups (p < 0.05 is considered statistically significant), Mann Whitney U-test.

### Occurrence of apoptosis in the livers of severe *P. falciparum* malaria cases

**Figure 3:**
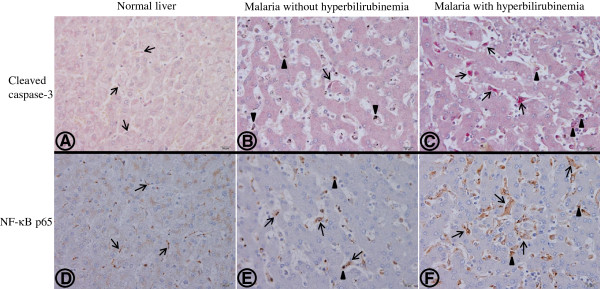
**Representative immunohistochemical staining patterns of cleaved caspase-3 (A-C) and NF-κB p65 (D-F) in a normal liver showing negative staining for hepatocytes and Kupffer cells (A,D); in the livers of a severe *P. falciparum *malaria case without hyperbilirubinaemia (B,D) and with hyperbilirubinaemia (C,F).** Hepatocytes are generally unaffected. Arrows show Kupffer cells lying next to the hepatic cord, within the sinusoidal area. In the normal liver, Kupffer cells are small, non-reactive and rarely express the cleaved caspase-3 marker **(A)** and show few NF-κB p65 **(D)** compared with severe *P. falciparum* malaria without hyperbilirubinaemia **(B,E)** and with hyperbilirubinaemia **(C,F)** where Kupffer cells are enlarged and hyperplastic. Most Kupffer cells containing haemozoin pigment (arrowheads) expressed apoptotic and NF-κB p65 markers. Numerous Kupffer cells show apoptosis **(C)** and NF-κB p65 expression **(F)** in severe *P. falciparum* malaria with hyperbilirubinaemia. All images are at 400x magnification; bars are 20 μm.

**Figure 4:**
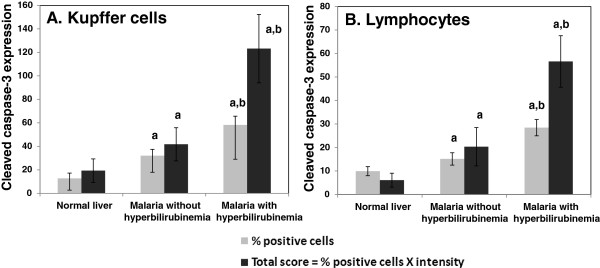
**Cleaved caspase-3 expression in Kupffer cells and lymphocytes in the portal tracts.** Significant differences were observed between the severe malaria groups (with and without hyperbilirubinaemia) and the normal controls (a), and between the hyperbilirubinaemia and non-hyperbilirubinaemia groups (b) (*p* < 0.05), in both percentage and total scores for cleaved caspase-3 expression in Kupffer cells **(A)** and lymphocytes in the portal tracts **(B)**. Data are presented as mean ± SEM.

Apoptosis was evaluated using monoclonal antibody against cleaved caspase-3, the final apoptotic pathway. The occurrence of apoptosis in the hepatocytes was negligible even in the severe *P. falciparum* malaria with hyperbilirubinaemia group. Figure 3 A-C illustrates the occurrence of apoptosis in the liver tissues of severe *P. falciparum* malaria cases, compared with the normal controls. Percentage apoptosis for Kupffer cells and lymphocytes within the portal tracts is shown in Figure 4. The data show that both Kupffer cell and lymphocyte apoptosis were significantly increased in group with severe *P. falciparum* malaria with hyperbilirubinaemia compared with the non-hyperbilirubinaemia groups (*p* = 0.030 and *p* = 0.009, respectively) and the normal controls (all *p* < 0.001) (Figure 4A and B). The Kupffer cells and lymphocytes in the non-hyperbilirubinaemia group also expressed significantly higher levels of apoptosis than the normal controls (*p* = 0.005 and *p* = 0.073, respectively). Since immunostaining intensity is an important factor in evaluating the degree of protein expression, a total score incorporating percentage apoptosis and immunostaining intensity was also used to compare apoptosis in the liver. The total scores for Kupffer cell and lymphocyte apoptosis are shown in Figure 4. A similar trend to the percentage data was observed.

### NF-κB p65 expression in the livers of severe *P. falciparum* malaria

**Figure 5:**
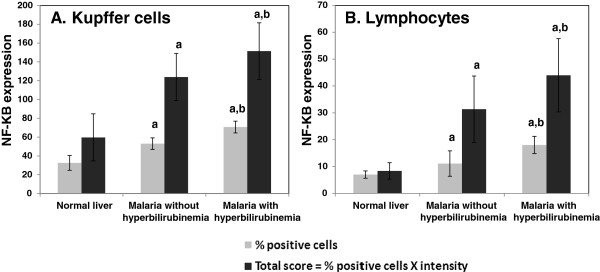
**NF-κB p65 expression in Kupffer cells and lymphocytes in the portal tracts.** Significant differences in percentage and total score for NF-κB p65 expression were observed in Kupffer cells **(A)** and lymphocytes in the portal tracts **(B)** between the severe malaria groups (with and without hyperbilirubinaemia) and the normal controls (a) and between the hyperbilirubinaemia and non-hyperbilirubinaemia groups (b) (*p* < 0.05). Data are presented as mean ± SEM.

NF-κB p65 expression and apoptosis in the livers of severe *P. falciparum* malaria were investigated. NF-κB p65 expression in Kupffer cells and portal lymphocytes is shown in Figure 5. In both Kupffer cells and lymphocytes, NF-κB p65 expression was significantly increased in the group with severe *P. falciparum* malaria with hyperbilirubinaemia, compared with non-hyperbilirubinaemia group (*p* = 0.030 and *p* = 0.009, respectively) and normal controls (all *p* < 0.001). The malaria group without hyperbilirubinaemia showed significantly higher levels of NF-κB p65 expression in the Kupffer cells and lymphocytes than the normal controls (*p* = 0.005 and *p* = 0.007, respectively).

### Correlation between apoptosis and NF-κB p65 expression

**Figure 6:**
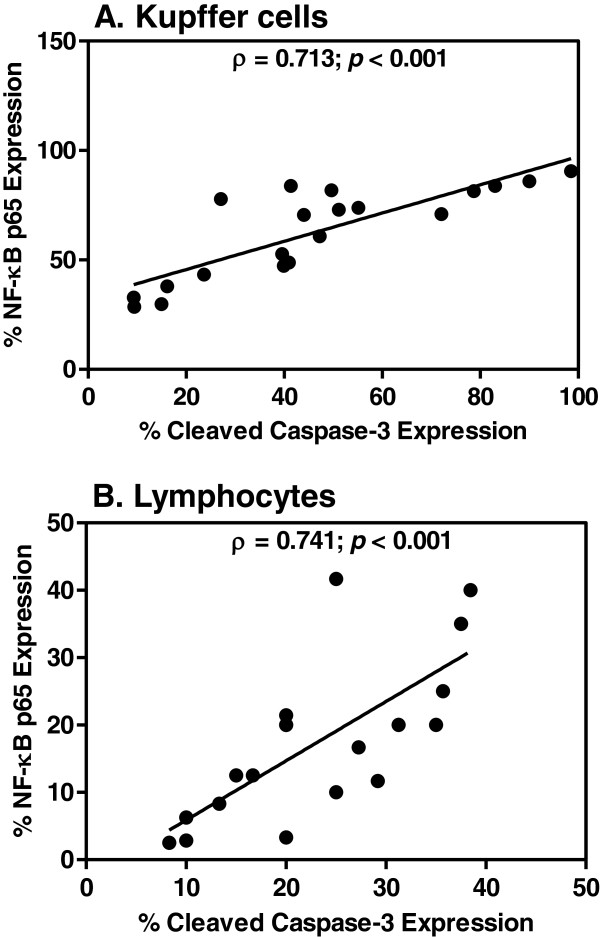
**Correlation between NF-κB p65 and cleaved caspase-3 activation. A-** in Kupffer cells (Spearman’s ρ test: *r*_*s*_ = 0.713, *p* < 0.001); **B-** in lymphocytes within the portal tracts (Spearman’s ρ test: *r*_*s*_ = 0.741, *p* < 0.001).

A significant positive correlation was found between cleaved caspase-3 and NF-κB p65 immunopositive cells in Kupffer cells (*r*_*s*_ = 0.713, *p* < 0.001), and lymphocytes in the portal tracts (*r*_*s*_ = 0.741, *p* < 0.001) (Figure 6). Both the expression of cleaved caspase-3 and NF-κB p65 were positively correlated with TB (Kupffer cells: *r*_*s*_ = 0.707, *p* = 0.001 and *r*_*s*_ = 0.853, *p* < 0.001, respectively) (lymphocytes: *r*_*s*_ = 0.490, *p* = 0.039 and *r*_*s*_ = 0.636, *p* = 0.008, respectively). Hyperplastic Kupffer cells were positively correlated with cleaved caspase-3 (*r*_*s*_ = 0.617, *p* = 0.001) and NF-κB p65 (*r*_*s*_ = 0.568, *p* = 0.001) expression. The degree of portal tract inflammation was also positively correlated with cleaved caspase-3 (*r*_*s*_ = 0.612, *p* = 0.001) and NF-κB p65 (*r*_*s*_ = 0.519, *p* = 0.002) expression. On the other hand, no significant association was seen between both NF-κB p65 and cleaved caspase-3 expression of Kupffer cells and portal lymphocytes, and clinical data (age, sex, days of fever pre-admission, parasitaemia and important liver function tests.

## Discussion

### Liver pathology in severe *P. falciparum* malaria

The present study shows a rise in liver transaminases and alkaline phosphatase in the malaria group with hyperbilirubinaemia (TB ≥ 51.3 μmol/L). Clinical jaundice in *P. falciparum* can be caused by several factors, i.e. intravascular haemolysis from parasitized red blood cells (PRBCs), G6PD deficiency-related haemolysis or anti-malarial drugs, disseminated intravascular coagulation (DIC) or co-existing septicaemia-induced hepatitis [14,15]. The histopathology of the liver in severe malaria has been previously studied. However, the present study demonstrated certain morphological variations in the liver from other reports, such as an abundant chronic inflammatory cell response and an absence of liver cell necrosis. Liver changes in severe malaria often include hyperplastic Kupffer cells [4,5,16-18], fatty change [16,17], portal tract inflammation [17], cholestasis [16,17], liver cell necrosis [4,16,18], sequestration of PRBCs and the deposition of haemozoin pigment [4,5,16,18]. The present study documented hyperplastic Kupffer cells with scattered haemozoin deposition and portal inflammation as the most common histological changes in the livers of severe *P. falciparum* malaria cases. The enlarged Kupffer cells were confirmed quantitatively using Image Tool software. The immune response in the liver to PRBCs primarily involves the activation of Kupffer cells. The recruitment and activation of Kupffer cells and macrophages in the spleen and bone marrow are important for the clearance of malaria parasites [19]. Portal tract inflammation consists mainly of lymphocytes and a few plasma cells (Table 4), in contrast with mild inflammation (portal and lobular lymphocytic infiltrates) reported earlier [5]. An acute inflammatory process involving neutrophils is not seen. The minimal fatty change noted here was similar to a previous finding [5]. Liver cell necrosis in *P. falciparum* was not a striking finding in this study. It has also been reported as a rare event in some other studies [5,18]. However, the incidence of hepatic necrosis may be as high as 41% [4] and severe cases of centrizonal necrosis have been documented [18]. This change has been reported to be secondary to suppression of bilirubin excretion by PRBCs or metabolic acidosis rather than hepatitis *per se*[14]. Sequestration of PRBCs is a common finding and depends on malaria parasite load.

### Apoptosis and NF-κB p65 in the liver of severe *P. falciparum* malaria cases

Among various cells evaluated in the liver tissue, Kupffer cells and inflammatory cells show significant apoptotic changes in severe *P. falciparum* malaria. Hepatocytes, bile ducts and ECs failed to show significant apoptotic changes. *P. falciparum* has been shown to induce apoptosis in human cells, such as in lymphocytes [13,20-22], neurons [13], glial cells [13], brain ECs [13] and lung ECs [23]. This may be responsible for clinical manifestations and progression to severe disease. In animal models, malaria-induced apoptosis was evident in astrocytes [24], lymphocytes [25], liver and spleen [8,25]. During the liver stage, however, hepatocytes are usually spared from the apoptotic process, allowing merozoites to be released into the circulation, suggesting that malaria sporozoites can block pro-apoptotic pathways [26]. The present study focuses on liver changes in the erythrocytic stage, which is far beyond the liver stage of parasite development. Nevertheless, hepatocytes remain morphologically unaffected and protected from apoptosis. Hepatocytes are defended by a barrier of Kupffer cells, endothelial cells, stellate cells, space of Disse (perisinusoidal space) and Kupffer cells. Moreover, PRBCs localized within the sinusoidal area are in close contact with Kupffer cells, the first-line immune defense in the liver. Recognized as foreign bodies, PRBCs and haemozoin are primarily engulfed by the Kupffer cells. In contrast, apoptosis in the hepatocytes has been reported in animal models, linked to activation of the mitochondrial pathway, release of reactive oxygen species [7,8] and induction by glycosylphophatidylinositol (GPI), a major membrane-associated protein of *P. falciparum*[27].

Apoptosis in the Kupffer cells was evidenced by strong caspase-3 expression. The loaded haemozoin within the cytoplasm of Kupffer cells can be toxic to these immune cells. In humans, during the erythrocytic stage, malaria parasites degrade haemoglobin to produce haem and haemozoin which are harmful. Haemozoin can be deposited in the liver and primarily phagocytized by Kupffer cells, where it can induce oxidative stress [7], a possible mechanism for the induction of apoptosis in Kupffer cells. During the liver stage, Kupffer cell apoptosis has also been detected in a murine model after incubation with *Plasmodium yoelii* sporozoites [9]. Apoptosis in malaria is reportedly mediated by the Fas-ligand in lymphocytes [20] and in murine astrocytes [24].

NF-κB has been shown to regulate various cellular processes such as inflammation, immunity, cell proliferation and apoptosis [28]. A previous study documented NF-κB activation and pro-inflammatory response in human brain ECs exposed to PRBCs [29]. A recent study of human brain tissues has demonstrated that NF-κB is one of the signaling molecules that modulates apoptosis in brain ECs and intravascular leukocytes in fatal cerebral malaria [13]. The present study documented NF-κB mediating apoptosis in Kupffer cells and lymphocytes within the portal tract in severe *P. falciparum* infection.

## Conclusions

Histopathological changes in the livers of severe *P. falciparum* malaria cases are associated with total bilirubin levels. Apoptosis of Kupffer cells and portal tract lymphocytes is a significant finding and is related to NF-κB activation.

## Competing interests

The authors declare that they have no competing interests.

## Authors’ contributions

PV initiated the research idea, designed the experiments, evaluated histopathology and immunohistochemistry work, supervised, and revised the manuscript. VK retrieved formalin-fixed specimens, performed the histopathology techniques and drafted the manuscript. CP participated in the study design, carried out the immunohistochemical work, preliminary data analysis and manuscript preparation. All authors have approved the final version of the manuscript.
